# Basal cutaneous pain threshold in headache patients

**DOI:** 10.1007/s10194-011-0313-9

**Published:** 2011-02-19

**Authors:** Maurizio Zappaterra, Simona Guerzoni, Maria Michela Cainazzo, Anna Ferrari, Luigi Alberto Pini

**Affiliations:** Headache and Drug Abuse Inter Dept. Research Centre, University of Modena, Modena, Italy

**Keywords:** Headache, Pain threshold, Allodynia, Central sensitization, Medicine overuse

## Abstract

The aim of this study was to analyze cutaneous pain threshold (CPT) during the interictal phase in headache patients, and the relationships between headache frequency and analgesic use. A consecutive series of 98 headache patients and 26 sex- and age-balanced controls were evaluated. Acute allodynia (AA) was assessed by Jakubowski questionnaire, and interictal allodynia (IA) by a skin test with calibrated monofilaments. AA is widely known as a symptom more present in migraine than in TTH spectrum: in our study this was confirmed only in cases of episodic attacks. When headache index rises towards chronicization, the prevalence of AA increases in both headache spectrums (*χ* ^2^ 13.55; *p* < 0.01). AA was associated with IA only in cases of chronic headache. When headache becomes chronic, mostly in presence of medication overuse, interictal CPT decreases and IA prevalence increases (*χ* ^2^ 20.44; *p* < 0.01), with closer association than AA. In MOH patients there were no significant differences depending on the diagnosis of starting headache (migraine or tension type headache) and, in both groups, we found the overuse of analgesics plays an important role: intake of more than one daily drug dramatically reduces the CPT (*p* < 0.05). Thus, when acute allodynia increases frequency, worsens or appears for the first time in patients with a long-standing history of chronic headache, it could reasonably suggest that the reduction of CPT had started, without using a specific practical skin test but simply by questioning clinical headache history. In conclusion, these results indicate that the role of medication overuse is more important than chronicization in lowering CPT, and suggest that prolonged periods of medication overuse can interfere with pain perception by a reduction of the pain threshold that facilitates the onset of every new attack leading to chronicization.

## Introduction

Allodynia is an abnormal sensory condition during which normally innocuous stimuli are felt as painful. Sensitivity changes, during headache attacks, have shown by common symptoms like, a feeling of discomfort when patients wear tight-fitting clothing, necklaces, glasses or when hair is bound; or, also, when exposed to excessive heat or cold. Migraineurs, during their attacks, highlight a decreased pain threshold to non-noxious thermal and mechanical stimulation of skin, frequently referred as cutaneous allodynia. Using quantitative and semi-quantitative sensory testing techniques, Burstein et al*.* have demonstrated the gradual development of cutaneous allodynia in subjects with episodic migraine after the onset of their migraine attacks [1–4].

Animal studies support this hypothesis, in fact, the application of an inflammatory soup to dura, in a rat model of intracranial pain, followed by an innocuous stimulus, generates a pronounced increase in neuronal firing patterns in peripheral and central second-order neurons of the trigemino-vascular system [5]. It has been suggested that the time course and anatomic progression of this hypersensitivity correlate with the time course of the development of central sensitization. So, in patients with episodic migraine, cutaneous allodynia is likely to represent a clinical correlate to the central sensitization.

Patients with frequent migraine usually evolve from episodic attacks; the possible pathogenetic explanation for this is that pain pathways become chronically sensitized from repeated episodes of headache attacks [6–8].

This phenomenon could help to explain the clinical observation that frequent episodic migraine develops into transformed migraine (TM), and should therefore require early prophylactic therapy to prevent the increase of headache frequency, that is related to the headache chronicization [6].

Moreover, given what has been shown to happen to pain threshold during episodic migraine, one might expect that patients with very frequent migraine would also have a lower basal pain threshold (in absence of headache) than normal subjects. Thus far, there have been no studies to determine whether the cutaneous pain threshold (CPT) is lowered during the interictal phase in patients who have chronic headache (more than 15 days per month) in comparison to episodic headaches and the headache-free population.

The aim of this study is to evaluate the relationship between CPT and headache clinical characteristic with regard to frequency, acute allodynia (AA), duration and analgesic overuse.

## Materials and methods

### Patient population

We enrolled 102 patients, but only 98 performed all tests and were evaluated for this study. All subjects were outpatients that were selected in the Headache and Drug Abuse Inter Department Research Centre at the University of Modena (Italy), after signing written informed consent, between December 2008 and July 2009. All data used were collected using patient’s medical history and diary cards recorded in clinical folders that are carefully kept in the Headache Centre. Patients who meted the inclusion criteria were sent to the ward where a trained doctor (Z.M.) filled clinical questionnaire and performed the CPT test. The CPT tests were performed in the morning. Patients with episodic headache were pain-free for at least 48 h before testing, and sufferers with chronic headache were tested in the morning before taking any drug.

Control subjects were recruited from hospital staff by the headache clinic nurse; these subjects were used rather than normative data because it was unlikely that the conditions of the normative trials could be exactly reproduced, and it has been suggested that normative data in pain threshold testing may be unreliable in different study populations.

For each patient we have analyzed the subsequent three parameters: (1) daily drug intake (DDI), number of drug doses consumed every day; (2) headache index (HI), number of days with headache attacks; (3) years of chronicization (y. CHR), the time elapsed since the beginning of chronicization. The daily drug intake and the headache index were referred to 3-month period before testing; while the period for chronicization was considered when lasting more than 1 year.

This study was performed following the Helsinki Declaration principles and approved by the University of Modena and Reggio Emilia regulatory authorities.

### Inclusion criteria

All patients were diagnosed by trained physicians according to the ICHD-II criteria [9]. The following abbreviations were used for the diagnoses: MIG = episodic migraine with and without aura (ICHD 1.1 and 1.2); ETTH = episodic tension type headache (ICHD 2.1 and 2.2); CTTH = chronic tension type headache (ICHD 2.3), MOH = medication overuse headache (ICHD 8.2). The MOH group was also divided in two sub-groups according to the type of starting headache and classified as TM-MOH (TM = transformed migraine, following Silberstein [10]) when first diagnosis was in the migraine spectrum, or TTH-MOH (TTH = tension type headache) when first diagnosis belonged to the tension type spectrum. We included in the control group age- and sex-matched healthy subjects.

### Exclusion criteria

Patients younger than 18 years, patients with a secondary headache, or headache associated to sensory loss/tingling, neuropathy; or also patients with history of narcotic-seeking behaviour, or, above all, patients unable to avoid analgesic medication 12 h before the cutaneous pain threshold test (CPT) were excluded from the study protocol.

### Allodynia questionnaire

#### Acute allodynia

In this study, this term is used and reported as a phenomenon that occurs during headache attack. AA was assessed following Jakubowski’s allodynia questionnaire [11], and this parameter was correlated with CPT and clinical features of headaches and control groups. Specific questions were used for confirming skin sensitivity in past attacks and, according to these, patients were classified either as allodynic (> or =1 symptom) or non-allodynic (zero symptoms).

### Cutaneous pain threshold test

In order to minimize bias, all patients and controls were recruited by the headache clinic nurse; all the CPT assessments were carried out during the interictal phase (in absence of headache attack) by one investigator (Z.M.). To investigate skin sensitivity, calibrated von Frey-like filaments (Touch-Test^®^ Sensory Evaluators filaments, NorthCoast Medical Inc., CA) were applied sequentially in increasing order for 2 s each to determine the initial (basal) CPT, asking the patient when the touch becomes a painful or very uncomfortable sting. Trials were repeated three times and the filaments were used in each location in the following calibration representing the respective, ascending forces in grams of each of the filaments (0.02, 0.04, 0.16, 1, 4, 6, 15, 60, 100, 180 and 300 g).

The test investigated three cutaneous areas: temple, cheekbone and cervical areas (exploring first, second and third divisions of the trigeminal nerve, respectively), assessed bilaterally in random order, to measure the sensitivity of skin outside the headache crisis.

Landmarks definition were: cervical, the skin over the cervical and trapezius muscles, between C2 and C4 level; cheekbone, the skin over periorbital area of zygomatic bone; temple, the skin over the pars orbitalis of frontal and sphenoid bones.

The test was always performed bilaterally out of crisis in episodic headache and in presence of low pain (value 1) in chronic headaches. In chronic patients we did not record a unilateral pain during test. In fact, we did not find any statistical difference either between sides in all groups, or between sex. Moreover, as showed in Table 1 there were no statistical differences within areas, so we decided to use the mean values as an indicator of a generic skin threshold of the head.

**Table 1:** Interictal mean CPT values recorded by CPT test with calibrated monofilaments

Group	Temple		Cheekbone		Neck	
	Mean ± SD (g)	*p*	Mean ± SD (g)	*P*	Mean ± SD (g)	*p*
CTRL	127.7 ± 75.6	NA	130.7 ± 76.1	NA	106.1 ± 59.5	NA
ETTH	70.3 ± 56.9	0.005	67.9 ± 56.9	0.004	80.7 ± 58.5	0.14
MIG	62.3 ± 72.6	0.004	61.6 ± 73.2	0.003	90.4 ± 91.2	0.49
CTTH	38.1 ± 55.7	<0.001	40.9 ± 58.2	<0.001	71.9 ± 60.2	0.13
MOH	19.7 ± 31.8	<0.001	12.6 ± 28.6	<0.001	29.4 ± 37.2	<0.001
TM-MOH	18.6 ± 23.2	<0.001	8.1 ± 11.6	<0.001	29.9 ± 42.7	<0.001
TTH-MOH	21.5 ± 42.9	<0.001	19.9 ± 43.5	<0.001	28.5 ± 27.6	<0.001

Student’s *t* test, *p* versus CTRL

To establish pathological value for CPT, we used a quantitative definition of allodynia, the same as used in other studies [1, 11, 12]. Since we measured basal skin sensitivity in the absence of headache, in cases of abnormal reduction of CPT, we decided to use the term “interictal allodynia” (IA). Using this “cut-off” method, individual patients were identified as having IA to mechanical stimuli when the three tested areas showed a mean overall CPT 1 or more standard deviation (SD) less than the basal mean CPT, in the same areas of control subjects.

Our study has some limitations as the choice of controls was from the hospital staff. In fact, these subjects could have more awareness on nociceptive tests and were younger as patients, but it was reasonable to perform the test in the same way in patients and controls than compare results with literature data. We are aware of the poor statistical power of so small a group of controls, but it is reasonable to use a control group to define cut-off values, unbiased between sites. The use of one SD as parameter was used by Burstein et al. [1], but it could be discussed.

### Statistical analysis

Continuous variables (age, y.CHR_,_ HI, DDI and CPT) were expressed as mean ± standard deviation. To compare CPT values between controls, headache groups and subcategory of headache patients’ features, we used independent unpaired samples (with unequal variances) *t* test and Bonferroni correction for multiple comparison; all *p* values were two-sided. A sample mean comparison test was used to indicate the normal CPT, useful to get an arbitrary quantitative definition of cutaneous IA. To compare the prevalence of AA and IA and estimate the degree of association with headaches and conditions, data were analyzed by Chi-Square Test of Association, Odds Ratio and, in case of low sample, by Fisher Exact Probability Test. To correlate CPT values with DDI, y.CHR and HI, we used the Pearson correlation test. One-way ANOVA for multiple comparison was used to compare CPT levels and type of medication. STATA software (version 10, StataCorp LP, TX, USA) was used to perform statistical analyses.

## Results

### Demographic data

98 patients aged between 19 and 65 years (mean ± SD = 44.4 ± 14.5); 72 F (aged 43.1 ± 14.4) and 26 M (aged 47.8 ± 14.7) were divided in to 4 main groups: ETTH, CTTH, MIG and MOH. The MOH group was divided in two subgroups: transformed migraine (TM), if starting headache was migraine or tension type headache (TTH), if initial headache was tensive.

The control group of 26 subjects was aged between 18 and 63 (40.2 ± 18.2); 9 M and 17 F, aged 43.9 ± 16.7 and 38.2 ± 19.1, respectively. There were no sex differences between male and female so data were omitted. Data are summarized in Table 2.

**Table 2:** Demographic data

Group	*N*	Age	Male (*N*)	Female (*N*)
CTRL	26	40.2 ± 18.2	9	17
ETTH	22	37.0 ± 11.4	7	15
MIG	21	38.2 ± 12.3	4	17
CTTH	11	46.6 ± 20.0	3	8
MOH	44	50.3 ± 12.8	12	32
TM-MOH	27	48.1 ± 24.4	8	19
TTH-MOH	17	35.4 ± 20.7	4	13

TM-MOH and TTH-MOH are subgroups of MOH

*MIG* episodic migraine with and without aura (ICHD 1.1 and 1.2), *ETTH* episodic tension type headache (ICHD 2.1 and 2.2), *CTTH* chronic tension type headache (ICHD 2.3), *MOH* medication overuse headache (ICHD 8.2), *TM-MOH* MOH started as migraine, *TTH-MOH* MOH started as tension type headache

### Allodynia

The percentages of patients resulting positive at the allodynia questionnaire (AA) and positive at the CPT (IA) are reported in Table 3.

**Table 3:** Clinical data

Group	*n*	HI	y.CHR	AA % (*n*)	IA % (*n*)
CTRL	26	–	–	– (0)	– (0)
ETTH	22	0.14 ± 0.15	–	18.2% (4)	27.2% (6)
MIG	21	0.19 ± 0.13	–	66.6% (14)	52.3% (11)
CTTH	11	0.82 ± 0.18	6.9 ± 6.7	81.8% (9)	72.7% (8)
MOH	44	0.88 ± 0.15	15.6 ± 13.6	77.2% (34)	86.4% (38)
TM-MOH	27	0.85 ± 0.34	17.4 ± 15.2	81.4% (22)	85.1% (25)
TTH-MOH	17	0.72 ± 0.42	3.3 ± 3.9	70.6% (12)	88% (15)

TM-MOH and TTH-MOH are subgroups of MOH. Numbers in brackets

*HI* headache index, *y.CHR* = years of chronicization, *AA* acute allodynia estimated by questionnaire, *IA* interictal allodynia evaluated by CPT test

A more detailed analysis showed that IA is closely associated with the chronic form of headache, more than AA [IA (OR = 7.82, 95% CI 3.42–23.64); AA (OR = 4.9, 95% CI 2.06–12.01)].

### Questionnaire results

Taking into account all patients, AA prevalence was higher in chronic (78.2%) than episodic headaches (41.8%) (*χ* ^2^ 13.55; *p* < 0.01). AA was more frequent in migraine form (75%) than in tension type form (50%) (*χ* ^2^ 7.25; *p* < 0.01). A deeper analysis showed that only in cases of episodic attacks AA prevalence was higher in migraine (66%) than in tension type headache (18%); (Fisher exact probability test *p* = 0.001). In chronic headache group (both CTTH and MOH) the difference of AA between TM-MOH (81%) and tension type headache (CTTH and TTH-MOH (75%) disappeared (see Table 3).

### Cutaneous pain threshold

Pathological values of CPT were assessed for each area: temples <56 g; cheekbone <52 g; neck <46 g, following the cut-off method, calculated as 1 SD lower than mean CPT in the control group. While there were no statistical differences between mean CPT values of the three tested areas, to estimate IA prevalence we assessed a pathological value (<56 g) for the whole generalized mean CPT of overall areas. This cut-off threshold value was statistically significant, analyzed and validated by one sample comparison *t* test (*p* < 0.01). Subjects were considered as having IA, if mean CPT of overall areas was lower than <56 g at the CPT.

Only chronic headaches showed pathological mean CPT values, and IA prevalence was higher in chronic headaches (83.6%) than episodic headaches (39.5%) (*χ* ^2^ 20.44; *p* < 0.01).

**Fig. 1:**
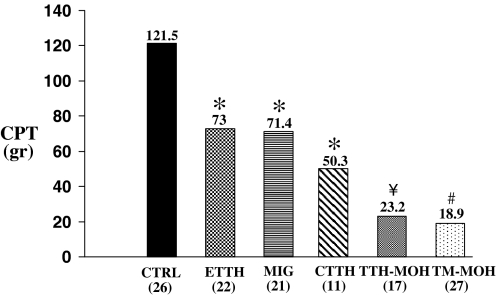
Mean CPT values resulting from the CPT test in all headache groups. (Student’s *t* test; *p* < 0.01; *vs. controls; ^¥^vs. controls, ETTH, MIG; ^#^vs. controls, ETTH, MIG, CTTH). In *brackets* number of cases

CPT values (mean ± SD) in the three tested areas of all headache groups and statistical significance versus CTRL (Student’s *t* test *p* value) are collected in Table 1. Figure 1 shows the overall areas of mean CPT values of CTRL and all headache groups, with significant statistical differences between groups. No statistical differences were found either between male and female or between sides (dx vs. lf), so data were omitted.

Mean CPT values of all headache groups were statistically more significantly reduced than CTRL, and mean difference between CTRL and patients groups ranged from 48.54 g (ETTH) to 102.87 g (TM-MOH). The MOH group presented the lowest absolute CPT values compared to every group, in every area (*p* < 0.01). ETTH, MIG and CTTH groups, compared to CTRL, showed significant reduced mean CPT only in temple and cheekbone areas (*p* < 0.01); while sensitivity of the neck area was similar to the CTRL group.

A more detailed evaluation was performed for the MOH group: no statistical differences were found between the two subgroups TM-MOH and TTH-MOH.

Taking into account all headache sufferers, there was a negative linear correlation between HI and CPT, with statistical significance (*r* = −0.43; *p* < 0.01). In fact, chronic headaches (26.5 ± 35.2 g), but not episodic headaches (72.2 ± 62.9 g), showed extremely reduced mean CPT values (*p* < 0.01).

The duration of chronicization is associated with CPT reduction: taking into account all chronic patients (CTTH and MOH, *n* = 55), we found a negative linear correlation between mean CPT and y.CHR (*r* = −0.431; *p* = 0.01). The mean CPT in each clinical headache group is reported in Fig. 1

**Fig. 2:**
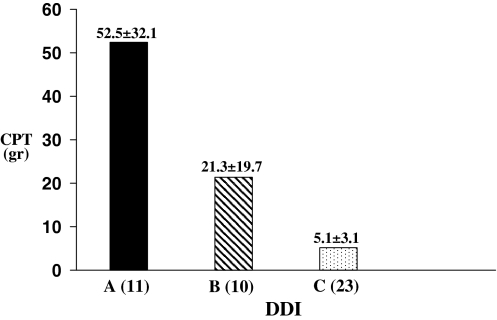
Mean ± SD of CPT reduction in MOH group, taking into account DDI divided in three subgroups. Group A (11): DDI ≤ 1; group B (10): DDI > 1, ≤ 2; group C (23): DDI > 2; (Bonferroni *t* test; *p < 0.05 vs. B and C). In *brackets* number of cases

The comparison between patients with and without drug abuse (CTTH and MOH groups) showed that presence of medication overuse played a role in CPT reduction, similar to chronicization. There was strong association of medication overuse with presence of IA (*χ* ^2^ 16.9; *p* < 0.01), stronger (approximately double) than the association with presence of AA (*χ* ^2^ 7.67; *p* < 0.01). In the whole MOH group, mean CPT values decreased depending on the number of DDI, with negative linear correlation (*r* = −0.436; *p* < 0.01). As shown in Fig. 2, patients who took more than one analgesic medication per day showed the lowest mean CPT values (Bonferroni Test: *p* < 0.05 A vs. B and C). Moreover, the mean CPT of patients with DDI equal to or less than 1 (52.5 ± 32.1 g) was lower than the CTRL group (*p* < 0.01), not being too far from normal value (>56 g).

**Fig. 3:**
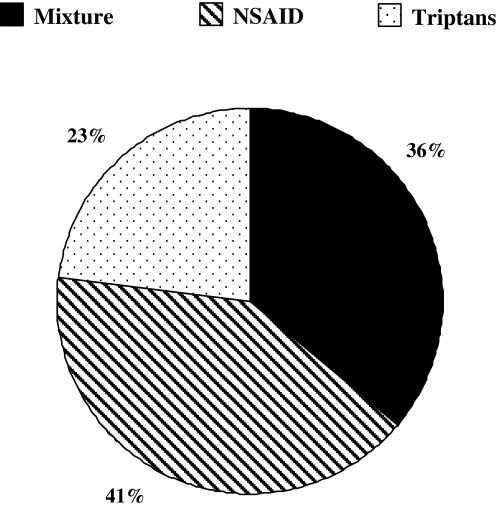
Prevalence of abused drugs in MOH group (*N* = 44)

There were three main groups of abuse, divided according to the type of primary abused drug: NSAID (*n* = 18; 41%), mixture drugs (*n* = 16; 36%) and triptans (*n* = 10; 23%) in Fig. 3. The mean DDI in the group of abusers (MOH) was 2.6 ± 1.8; the highest DDI rate was for mixtures 3.42 ± 3.03 followed by triptans 2.60 ± 1.76 and then by NSAID 1.97 ± 1.11, but no statistical differences were found within these groups on DDI (Bonferroni *t* test).

We also analyzed the relation between CPT and the types of analgesic drugs overused by patients. The lowest mean CPT was for mixture (13.8 ± 17.5 g), followed by triptans (19.44 ± 21.46 g) and then by NSAID (27.3 ± 34.7 g) but, probably due to the paucity of data, no statistically significant differences were found in mean CPT between the different types of primary abuse associated with the MOH group.

### Relationships between AA, IA and CPT

To investigate the relationship between AA and IA, we compared the results of the two tests (questionnaire and CPT) across the different groups and features of headaches.

In cases of episodic headaches, there was no significant difference between mean CPT values depending on the presence (57.2 ± 70.3 g) or the absence (83 ± 55.9 g) of AA, both with normal interictal skin sensitivity.

Only in cases of chronicization did subjects with AA (21.4 ± 33.2 g) show lower mean CPT than subjects without AA (44.6 ± 37.6 g) and both showed allodynic mean CPT values (*p* < 0.05).

The rate of simultaneous presence of both AA and IA was higher in chronic than episodic headaches (*χ* ^2^ = 16.4; OR = 5.77.95% CI 2.4–13.9; *p* < 0.0005).

## Discussion

The primary objective of this study was to estimate pain threshold during the interictal phase, in different types of episodic and chronic headaches, comparing four groups of headache patients with a sex- and age-matched control group. We assessed, also, the prevalence of allodynia during attacks and recorded CPT in scheduled cranial-facial areas outside headache crisis, to determine if they were altered, and to correlate the variation of this parameter to the time course of the headache itself.

Epidemiological studies identify the following major risk factors for worsening of migraine: frequency of headache attacks at baseline, obesity, medication or caffeine overuse, sleep disorders (including sleep disordered breathing), stressful life events and depression [13–17].

Although the first neurological event leading to migraine pain is still not completely understood, it has been suggested that dysfunction of the brainstem involved in the modulation of craniovascular afferents may lead to activation of ascending and descending pathways with onset of a perimeningeal vasodilatation and neurogenic inflammation [18].

Preclinical studies show that drug abuse can induce pro-nociceptive neuroadaptive changes in the oro-facial division of the trigeminal ganglia that persist even after discontinuation of drug treatment. Additionally, medication abuse can elicit an increase of descending facilitatory influences that may amplify inputs from trigeminal afferents leading to behavioural hypersensitivity clinically evident as cutaneous allodynia [19, 20].

Prevalence of AA, as previously reported in other studies, was higher in patients with MIG (66.6%) than ETTH (18.2%), but this difference was present only in episodic headache. When headache becomes chronic, the differences disappear: TM-MOH (81%) and chronic form of TTH (75%) (CTTH and TTH-MOH) showed similarly high percentages of AA. Following the Jakubowski allodynia questionnaire we found comparable prevalence as in literature, whereas we did not find difference between sex [1, 12, 21, 22] (see Table 3). These observations are confirmed by a prior study, performed using a specific questionnaire sent by post to a random sample of 24,000 headache sufferers, previously identified from the population, in which Bigal showed that the prevalence of acute allodynia was significantly higher in transformed migraine (TM = 68.3%) than in episodic migraine (63.2%, *p* < 0.01) and that it is more common and more severe in migraine and transformed migraine than in other primary headaches [21].

In our study we found that the skin sensitivity (CPT) in patients with episodic headache rises only during headache attack (AA), after which it reverts to normal values. Patients with MIG and ETTH showed similar mean CPT values during the interictal phase.

An increase of the interictal skin sensitivity (IA) was evident in chronic headache. In fact, while in episodic headache, patients with and without AA showed normal and similar mean CPT values, in chronic headache, patients with AA showed lower levels of mean CPT than those without AA. So, the AA may be suggestive of interictal CPT reduction only in case of chronicization (see Table 3).

When episodic headaches start AA may occur without IA. The contemporary presence of AA and IA were found only in chronic headache, especially in patients with medication overuse.

The chronicization in patients that suffer of headache modifies the pattern of headache itself, the sensitivity and the transmission of pain.

Pain is not only the expression of a nociceptive input, but the perception of balanced peripheral and central stimuli, often affected by emotional and cognitive influences [23–25].

This is more evident if the descending pain modulatory system is affected; in fact, the repetitive activation of trigeminovascular neurons reduce the activity of the periaqueductal gray (PAG) that is part, together other brain regions (frontal lobe, anterior cingulate cortex, amygdala, hypothalamus, rostral ventromedial medulla) of the anatomical network able to regulate nociceptive processing; silent brain damage and increase in iron concentration in deep nuclei are more frequent in patients with chronic migraine [26, 27].

Our results underline that high frequency of headache attacks task a significant reduction of CPT; CPT decrease if chronic headache lasts for some years, at least, 5–10 years. After this period CPT level remains constantly low. The pain temporal threshold is reduced in patients with medication overuse headache and improves after medication overuse was discontinued [28]

The role of medication overuse/abuse in reducing pain threshold has been suggested in previous studies [21, 22]. In these patients the link between pain and homeostasis is modified, as confirmed by observation that the vanilloid receptors, TRV1, cation channels that act as a polymodal detector of pain producing stimuli like capsaicin and protons (pH < 5.7), are tonically activated [29]. The central latent sensitization induced by the continuous increase in drug consumption could provide a mechanistic basis for evolution of migraine in medication overuse headache [4, 19, 30, 31].

In our data we have found a close connection between the number of analgesics used daily and the CPT (Fig. 2), whereas our limited sample does not allows us to draw conclusions about the effect on CPT of different analgesics used.

In conclusion medication overuse plays a role in lowering CPT more than headache chronicization itself, but further studies are needed. A prolonged period of medication abuse may increase the incidence of CSD events, or consequence of CSDs, to initiate signalling in the trigeminovascular system to enhance the frequency of migraine headaches, and, consequently, to promote persistent cephalic and extracephalic cutaneous allodynia [32].

It is difficult to discriminate the influence of length of chronicization and the possible effect of anti-migraine drugs. There are a number of studies on this argument, but there are not evidences that separate the chronicization from the activity of analgesic drugs. In our study we reported these new data: it seems that independently from patient’s histories, the most used drugs and concomitant factors (both psychological and physical), the association between chronic headaches and daily drug use lowered the CPT.

Finally, we are aware that one of the major limitations of this study is due to the paucity of the CTTH group and the lack of psychological pattern of drug abuser. Chronic patients without medication overuse are infrequent and a wider group could be really helpful to quantify the difference between the importance of medication overuse and chronicization in causing CPT reduction.
